# EBV T-cell immunotherapy generated by peptide selection has enhanced effector functionality compared to LCL stimulation

**DOI:** 10.3389/fimmu.2024.1412211

**Published:** 2024-07-01

**Authors:** Rachel S. Cooper, Catherine Sutherland, Linda M. Smith, Graeme Cowan, Mark Barnett, Donna Mitchell, Colin McLean, Stuart Imlach, Alan Hayes, Sharon Zahra, Champa Manchanayake, Mark A. Vickers, Gerry Graham, Neil W. A. McGowan, Marc L. Turner, John D. M. Campbell, Alasdair R. Fraser

**Affiliations:** ^1^ Tissues, Cells and Advanced Therapeutics, Scottish National Blood Transfusion Service, Jack Copland Centre, Heriot Watt Research Park, Edinburgh, United Kingdom; ^2^ Chemokine Research Group, Institute of Infection, Immunity and Inflammation, University of Glasgow, Glasgow, United Kingdom; ^3^ Institute of Immunology and Infection Research, School of Biological Sciences, University of Edinburgh, Edinburgh, United Kingdom; ^4^ Blood Transfusion Centre, Scottish National Blood Transfusion Service, Aberdeen, United Kingdom; ^5^ Microbiology and Immunity, School of Medicine, Medical Sciences and Nutrition, Institute of Medical Sciences, Aberdeen, United Kingdom

**Keywords:** cell therapy, Epstein-Barr virus, immunotherapy, T cell, potency, peptide, lymphoblastoid cell line, T cell receptor

## Abstract

Adoptive immunotherapy with Epstein–Barr virus (EBV)-specific T cells is an effective treatment for relapsed or refractory EBV-induced post-transplant lymphoproliferative disorders (PTLD) with overall survival rates of up to 69%. EBV-specific T cells have been conventionally made by repeated stimulation with EBV-transformed lymphoblastoid cell lines (LCL), which act as antigen-presenting cells. However, this process is expensive, takes many months, and has practical risks associated with live virus. We have developed a peptide-based, virus-free, serum-free closed system to manufacture a bank of virus-specific T cells (VST) for clinical use. We compared these with standard LCL-derived VST using comprehensive characterization and potency assays to determine differences that might influence clinical benefits. Multi-parameter flow cytometry revealed that peptide-derived VST had an expanded central memory population and less exhaustion marker expression than LCL-derived VST. A quantitative HLA-matched allogeneic cytotoxicity assay demonstrated similar specific killing of EBV-infected targets, though peptide-derived EBV T cells had a significantly higher expression of antiviral cytokines and degranulation markers after antigen recall. High-throughput T cell receptor-beta (TCRβ) sequencing demonstrated oligoclonal repertoires, with more matches to known EBV-binding complementary determining region 3 (CDR3) sequences in peptide-derived EBV T cells. Peptide-derived products showed broader and enhanced specificities to EBV nuclear antigens (EBNAs) in both CD8 and CD4 compartments, which may improve the targeting of highly expressed latency antigens in PTLD. Importantly, peptide-based isolation and expansion allows rapid manufacture and significantly increased product yield over conventional LCL-based approaches.

## 1 Introduction

Epstein–Barr virus (EBV), prevalent in over 90% of adults worldwide, was the first virus found to induce human malignancies (1). Like other human herpesviruses, EBV establishes a lifelong latency in the host. Most individuals acquire EBV early in life from salivary contact with carriers, after which it persists latently within B cell and mucosal epithelial cell reservoirs (2). Intermittent viral reactivation in B cells can trigger development into proliferative EBV-transformed lymphoblasts, usually controlled by EBV virus-specific T cells (VST). However, if unchecked, these transformed cells can develop into immunoblastic lymphomas. Immunosuppressed patients are at a particularly high risk of developing post-transplant lymphoproliferative disorder (PTLD) (3). Standard treatments for PTLD include reduction in immunosuppression, rituximab, and chemotherapy. If the PTLD is refractory to these treatments, the prognosis is very poor (4).

Adoptive transfer of donor EBV VST has proven to be an effective immunotherapy for relapsed or primary refractory PTLD patients (5). Several banks of cryopreserved allogeneic EBV VST have been developed for administration in a rapid “off-the-shelf” manner based on human leukocyte antigen (HLA) matching. Previously, we reported the first multicenter phase 2 trial to treat PTLD patients with allogeneic EBV VST, showing a 52% response rate at 6 months (6). Lower response rates were associated with hematopoietic transplant (46%) compared to solid organ transplant (75%) PTLD patients in a long-term follow-up study (7).

These EBV VST were generated using a conventional lymphoblastoid cell line (LCL) stimulation approach. Briefly, healthy donor mononuclear cells (MNC) were cultured with supernatant containing live EBV to transform B cells into LCL expressing viral lytic and latency proteins. The LCL were then irradiated and co-cultured with autologous MNC to stimulate the proliferation of VST specific for these viral proteins. Numerous LCL stimulation rounds are required to induce substantial EBV VST expansion (8). Despite production enhancement (9), this approach remains suboptimal in terms of clinical manufacture predominantly by biosafety aspects of using live virus to generate LCL and long culture durations.

Other techniques have been developed to derive VST, including isolation based on the secretion of cytokines in response to EBV peptide stimulation (10, 11). EBV VST have been isolated from donor blood stimulated with EBV peptide pools followed by a selection of interferon-gamma (IFN-γ)-secreting VST (12–14). This method provides a rapid isolation to administration approach (<36 h), but the low frequency of circulating EBV VST (~1% of total T cells) yields sub-optimally low numbers of IFN-γ+ target cells, thus restricting the dosing regimen. Protocols to generate cytomegalovirus VST have combined IFN-γ direct selection with a short culture to expand to clinically relevant numbers (15–17).

Here we describe the isolation of EBV-VST using good manufacturing practice (GMP)-grade EBV peptide pools followed by a rapid culture expansion, which results in high clinical product VST yields. Comprehensive characterization and comparison of final product EBV-VST generated by peptide IFN-γ selection versus LCL repeated stimulation revealed significant differences in T-cell memory, effector responses, and T-cell receptor (TCR) repertoire, which may contribute to potency and clinical efficacy of this immunotherapy.

## 2 Materials and methods

### 2.1 EBV VST manufacture

#### 2.1.1 Donors

Peripheral blood mononuclear cells (PBMC) for the LCL process manufacture were obtained by leukaphereses from blood donors in New Zealand, supplied cryopreserved by the New Zealand Blood Service and then transported to Scotland for storage in liquid nitrogen (LN_2_) until clean room manufacture. For the peptide-stimulated process, donation from UK donors was collected through leukapheresis via 5L Optia process with subsequent manufacture (within 72 h from donation) in European (EU) Grade B/C cleanroom facilities. All donors were fully consented, pre-screened for EBV sero-positivity, and had blood group O. The donors were also required to screen negative for the mandatory markers of infection/human immunodeficiency virus (HIV), human T-lymphotropic virus-1 (HTLV1), hepatitis B virus (HBV), hepatitis C virus (HCV), and syphilis.

#### 2.1.2 LCL repeated stimulation method

EBV VST were manufactured using the LCL repeated stimulation method as outlined in Vickers et al. (18). Briefly, donor MNC were infected with EBV B958 supernatant and cultured for 4–8 weeks until LCL developed. LCL were irradiated and co-cultured with remaining autologous MNC at an initial 30 MNC/1 LCL ratio. At day 10 and weekly thereafter, cultures were re-stimulated using 4 VST/1 LCL. The cultures were maintained in Roswell Park Memorial Institute (RPMI) medium containing irradiated fetal bovine serum (FBS; 20%) and interleukin-2 (IL-2; 20 IU/mL) for approximately 2 months. The cultures were tested after six stimulation rounds for minimal specificity in a flow cytometric cytotoxicity assay (8), and if >10% specific killing was observed, the cells were cryopreserved in Hank’s balanced salt solution (HBSS) containing human serum albumin (HSA; 10%) and dimethyl sulfoxide (DMSO; 10%) at 50 or 150 × 10^6^ cells per bag for storage in LN_2_. The final product was required to test negative for bacterial, mycoplasma, viral, and endotoxin levels prior to clinical use. VST release criteria also included <2% CD19+ B cells and >80% viability.

#### 2.1.3 Peptide-mediated IFN-γ selection and expansion method

For the peptide-stimulated method, potential donors’ peripheral blood PBMC were screened for T-cell responses to EBV consensus peptide pools using a flow cytometric cytokine assay, using a minimum criterion of >0.08% CD3+/IFNγ+ for a donor to be eligible for manufacture. Leukaphereses were volume-adjusted with acid citrate dextrose (ACD) buffer (Haemonetics or Fresenius Kabi) and IFN-γ-responsive T cells isolated using cytokine capture selection (CCS) on CliniMACS Prodigy (Miltenyi Biotech) using stimulation with GMP-grade EBV consensus peptide pools (Miltenyi Biotech). The peptide pools consisted of 43 MHC class I- and II-restricted peptides covering 15 EBV proteins: LMP1, LMP2A, EBNA1, EBNA2, EBNA3, EBNA4, EBNA6, BALF2, BRLF1, BMLF1, BMRF1, BNRF1, BZLF1, BLLF1, and BXLF2. Non-target cells were then irradiated (40 Gy) and co-cultured with IFNγ+ target cells at 1 IFNγ+ target cell to 200–400 irradiated non-target cells to drive expansion via continued co-stimulation and support in culture. Co-cultures were established in G-Rex100M-CS (closed-system) flasks in GMP-grade TexMACS serum-free medium (Miltenyi Biotech), supplemented with GMP-grade IL-2 (200 IU/mL; Cytiva) and expanded for 18–20 days with counts and feeds every 3 to 4 days. The cells were then harvested and cryopreserved at 150 × 10^6^ cells/bag in [2:1] Plasma-Lyte 148 (Baxter Healthcare Ltd) to CryoStor CS10 (BioLife Solutions) for LN_2_ storage. The final product was required to pass bacterial, mycoplasma, viral, and endotoxin testing as negative or not detected. VST were tested with a suite of quality control assays, with criteria of ≥90% CD3+ T cells, ≤10% CD3-/CD56+ NK cells, and ≥80% viability.

### 2.2 Phenotypic and functional characterization

#### 2.2.1 Immunophenotyping

For immunophenotyping, cryopreserved EBV VST were thawed, washed, and washed in PBS with EDTA (2.5 mM) and HSA (0.5%) (PEA buffer) with Fc receptor blocking reagent (Miltenyi Biotech). Cells were labeled with different antibody panels to assess T-cell memory and exhaustion. For chemokine receptor profiling, cells were labeled with anti-CD4, -CD8, and individual chemokine receptor antibodies (see **Table 1** for details of antibodies and panels). The cells were then washed and labeled with viability dye DRAQ7 (BioLegend) prior to acquisition on MACSQuant10 Analyser or BD Canto, recording 80,000 events. Flow cytometric analyses were gated as shown in **Supplementary Figure S1**, with populations classified in **Supplementary Methods**.

**Table 1:** Antibody details for immunophenotyping assays.

Panel	Marker	Clone	Conjugate	Surface or intracellular	Manufacturer
T-cell memory	CD45RA	T6D11	VioBlue	Surface	Miltenyi Biotec
	CD8	REA734	VioGreen	Surface	Miltenyi Biotec
	CD62L	145/15	FITC	Surface	Miltenyi Biotec
	CD3	BW264/56	PE	Surface	Miltenyi Biotec
	CD45RO	UCHL1	PerCP-Vio700	Surface	Miltenyi Biotec
	CD4	VIT4	PE-Vio770	Surface	Miltenyi Biotec
	CD56	AF12–7H3	APC	Surface	Miltenyi Biotec
T-cell exhaustion	LAG-3	11C365	BV421	Surface	BioLegend
	CD8	REA734	VioGreen	Surface	Miltenyi Biotec
	TIM-3	F38–2E2	FITC	Surface	BioLegend
	PD-1	REA1165	PE	Surface	Miltenyi Biotec
	CD4	VIT4	APC	Surface	Miltenyi Biotec
Chemokine receptor	CCR1	KF10B29	APC	Surface	BioLegend
	CCR2	K036C2	BV421	Surface	BioLegend
	CCR3	5E8	PE	Surface	BioLegend
	CCR4	L291H4	BV421	Surface	BioLegend
	CCR5	J418F1	BV421	Surface	BioLegend
	CCR6	G034E3	PE	Surface	BioLegend
	CCR7	G043H7	FITC	Surface	BioLegend
	CXCR3	G025H7	FITC	Surface	BioLegend
	CXCR4	12G5	PE	Surface	BioLegend
	CXCR5	J252D4	BV421	Surface	BioLegend
	CXCR6	K041ES	APC	Surface	BioLegend
	CD4	VIT4	PE-Vio770	Surface	Miltenyi Biotec
	CD8	REA734	VioGreen	Surface	Miltenyi Biotec
Intracellular cytokine and degranulation	IFN-γ	REA600	VioBlue	Intracellular	Miltenyi Biotec
	Granzyme B	GB11	BV510	Intracellular	BioLegend
	Perforin	dG9	BV711	Intracellular	BioLegend
	TNF-α	cA2	FITC	Intracellular	BioLegend
	CD107a	REA792	PE	Pre-stim	Miltenyi Biotec
	CD4	VIT4	PerCP-Vio700	Surface	Miltenyi Biotec
	IL-2	MQ1–17H12	PE-Cy7	Intracellular	BioLegend
	CD154	REA238	APC	Intracellular	Miltenyi Biotec
	CD8	SK1	AlexaFluor700	Surface	BioLegend

#### 2.2.2 Intracellular cytokine release degranulation assay

Cryopreserved EBV VST were thawed, washed, and plated at 2.5 × 10^6^ cells/cm^2^ in TexMACS. The following peptide stimuli were tested: individual EBV antigen pepmixes BARF1, BMLF1, BMRF1, BRLF1, BZLF1, EBNA1, EBNA2, EBNA3A, EBNA3B, EBNA3C, EBNALP, gp350/340, LMP1, and LMP2 (all JPT) and EBV consensus peptides (Miltenyi Biotech), with positive (PMA/ionomycin) and negative controls (no antigen). Cells were cultured overnight, and on the next day antibody CD107a was added. The peptides were added at 1 µg/mL for 5 h, with Brefeldin A (BioLegend) for the final 3 h, and wells were harvested for staining. Briefly, the cells were washed and labeled as detailed above, plus fixable viability dye (FVD) eFluor780. The cells were then fixed, permeabilized, and then labeled with intracellular antibody cocktail (see **Table 1**). The cells were analyzed on BD LSR Fortessa, recording 80,000 events.

#### 2.2.3 Cytotoxicity assay

Thawed VST were rested overnight and then assessed in a flow cytometric cytotoxicity assay. HLA-matched and mis-matched target LCL lines were labeled with PKH67 membrane dye (Sigma Aldrich) and then plated at 2.5 × 10^6^ cells/cm^2^ in triplicate per condition. LCL were cultured with VST at target to effector (T:E) ratios of 1:1, 5:1, 10:1, and 20:1—LCL only and T cell only. After 5 h, the cells were harvested and labeled with annexin-V-BV421 and dead cell dye DRAQ7 (both BioLegend). The cells were analyzed on MACSQuant10 flow cytometer. Cells were gated using PKH67+ to identify LCLs and analyzed for annexin-V and DRAQ7 positivity. To calculate the percentage-specific lysis at each T:E ratio, cytotoxicity was normalized to LCL-only wells. Evaluation of cytotoxic potency between lines was made by calculating the area under the curve of specific lysis over all T:E ratios from HLA-matched targets.

#### 2.2.4 Taqman low-density arrays

RNA was extracted from thawed VST using Qiagen RNAeasy columns with DNASe incubation. cDNA was synthesized using SuperScript VILO reverse transcription kits (Invitrogen), then diluted, mixed 1:1 with TaqMan Fast Advanced MasterMix, and run on a custom 384-well TaqMan low-density array (TLDA) card (Applied Biosystems) measuring the chemokine receptor, cytokine, transcription factor, senescence, and CD marker genes. Taqman low-density array (TLDA) cards were run on ABI Viia7 and analyzed for 2^-ΔΔCT^ in relation to RPLPO housekeeper gene and normalized against naïve CD3+/CD62L+/CD45RO- T cells isolated from buffy coats (*n* = 6).

#### 2.2.5 TCRβ next-generation sequencing and repertoire analysis

Samples were taken from donor CD3+ MNC and EBV VST final products. cDNA synthesis optimized for TCRβ amplification was performed as outlined (19). Subsequently, two rounds of PCR amplification were used to generate TCRβ variable region amplicons using indexed forward primers composed of the SMART oligo sequence with a P7 Illumina tag and P5 Illumina-tagged reverse primers within the TCR constant region (see **Supplementary Methods** and **Supplementary Figure S2**). PCR products were purified, and 2 × 300bp sequencing of libraries on the Illumina MiSeq platform were performed by GeneWiz using custom read primers (**Table 2**). Paired reads were initially assembled from FASTQs with PEAR (20) v0.9.6. Unique molecular identifier (UMI) processing, error correction, and VDJ assignment were carried out using Recover TCR (RTCR) v0.5.1 (21). RTCR output was processed in Python, with statistical analyses performed using the SciPy (22) and Pingouin (23) packages. Sequences sharing the same CDR3 amino acid sequence were considered to form a single clonotype, and repertoires were downsampled to the same number of UMIs. To identify TCR sequences of known specificity, CDR3s were queried against the Immune Epitope Database (IEDB) (24) using TCRMatch (25), and manually against VDJdb (26). The full list of public EBV-specific CDR3 sequences from Huisman et al. (27) was downloaded, and exact matches within VST repertoires were identified. Generation probabilities were calculated using Optimized Likelihood estimate of immunoGlobulin Amino acid sequences (OLGA) (28).

**Table 2:** Primers used for sample preparation and sequencing.

Step	Primer	Application	Sequence
cDNA synthesis	BC1R	cDNA synthesis	CAGTATCTGGAGTCATTGA
cDNA synthesis	SMART-NNN	Template switch	AAGCAGUGGTAUCAACGCAGAGUNNNNUNNNNUNNNNUCTTrG_(3)_
PCR 1	Smart_stepout1	Nested forward 1	CACTCTATCCGACAAGCAGTGGTATCAACGCAG
PCR 1	BC2R	Nested reverse 1	TGCTTCTGATGGCTCAAACAC
PCR 2	P7-SMART-Index	Forward	CAAGCAGAAGACGGCATACGAGATXXXXXXGGCGAAGCAGTGGTATCAACGCAGAGT
PCR 2	P5-BCJ	Reverse	AATGATACGGCGACCACCGAGATCTACACACACSTTKTTCAGGTCCTC
Sequencing	TCR_read1	Read 1	CGAG+ATCTACAC+ACACSTTKTTC+AGGTCCTC
Sequencing	TCR_read2	Read 2	GGCGAAGCAGTG+GTATCAACGCAGAGT
Sequencing	TCR&BCR index	Read index	ACTCTGCGTTGATACCACTGCTTCGCC

### 2.3 Statistical analysis

Statistical analyses were performed using Prism v9.4.1 (GraphPad). VST generated by LCL vs. peptide method were compared using unpaired *t*-tests corrected for multiple comparisons, with *p* < 0.05 considered statistically significant.

## 3 Results

### 3.1 Peptide-mediated IFN-γ selection method yields a high expansion of multifunctional EBV VST

**Figure 1:**
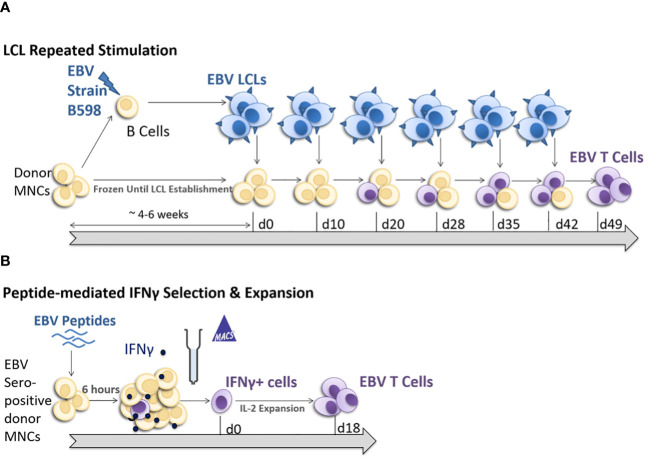
EBV-specific T-cell generation process comparison. In the conventional lymphoblastoid cell line (LCL) repeat stimulation process **(A)**, a portion of the donor mononuclear cells (MNC) was cultured in a medium containing Epstein–Barr virus (EBV) strain B598 supernatant for 4 6 weeks to induce the transformation of B cells into EBV-transformed LCL. The LCL are cryopreserved and then co-cultured with an autologous donor MNC over multiple stimulation rounds to drive the EBV antigen presentation to T cells, resulting in a highly enriched expanded EBV-specific T-cell product after six to eight rounds of stimulation. **(B)** For the peptide-mediated interferon-gamma (IFN-γ) selection and expansion process, MNC from EBV sero-positive donors were stimulated with EBV consensus overlapping peptide pools for 6 h, and reactive EBV memory T cells were isolated using IFN-γ cytokine capture selection. The non-target fraction from the selection is irradiated and co-cultured with the virus-specific target T cells to provide co-stimulatory support and T-cell expansion, resulting in rapid expansion to generate a highly enriched EBV-specific T-cell product within 18 days.

Treatment of EBV-induced lymphomas with therapeutic allogeneic EBV VST relies upon a fast, safe, and efficient manufacturing process that generates potent targeted products to ensure optimal patient responses and safety. We established two allogeneic EBV-VST banks using LCL stimulation (**Figure 1A**) that have treated ~200 patients with EBV-driven lymphomas to date. More recently, we developed a new manufacturing process to generate EBV VST using peptide-mediated IFN-γ selection with subsequent rapid culture expansion (**Figure 1B**).

**Figure 2:**
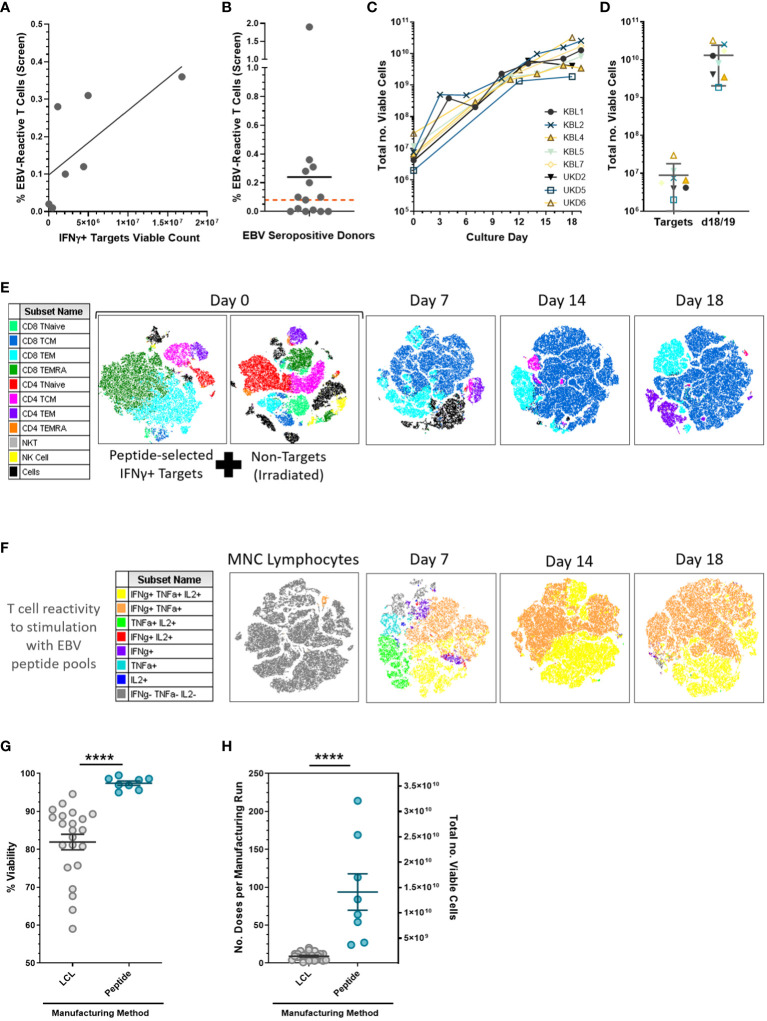
EBV-specific T cell peptide-mediated isolation and expansion. **(A)** Peptide-mediated selection requires a minimum threshold of EBV-reactive T-cell levels (frequency quantified using intracellular cytokine staining assay) to isolate sufficient viable IFN-γ targets for a Prodigy-based selection for full-scale manufacture (*n* = 7 development donor runs). **(B)** EBV sero-positive donors were pre-screened using peripheral blood samples for virus-specific T-cell responses where donors with a frequency of EBV peptide-reactive T cells (CD3+/ IFN-γ+) ≥0.08% of total lymphocytes (red threshold line); in total, 8/14 screened donors were suitable for manufacture. Data is presented as mean. **(C)** Growth curves from the full-scale production process demonstrated a two to three log expansion of EBV-specific T cells over 2 to 3 weeks of culture. **(D)** The mean ± SD viable cell count is shown for viable IFN-γ targets, and on days 18/19 the final products were harvested. **(E)** t-SNE analysis of multi-parameter flow cytometric surface marker phenotyping using representative donor UKD6 identified lymphocyte subpopulations from the selection and indicated an outgrowth of CD8+ central memory cells throughout the culture period. **(F)** Final product T cell reactivity to EBV consensus peptide pools (from the intracellular cytokine assay) showed a huge enrichment of the circulating EBV memory T cells from the starting material (MNC lymphocytes) to post-Prodigy selection (day 7) and subsequently through further expansion by days 14–18 culture. Peptide-derived EBV VST lines (*n* = 8) showed a significantly increased final product **(G)** viability and **(H)** yield as measured by the number of patient doses (1.5 × 10^8^ cells per dose) per manufacturing run on the left y-axis and total number of viable cells on the right y-axis compared to LCL-derived EBV VST lines (*n* = 22). Data is presented as mean ± SEM, and statistical analysis was performed using unpaired *t*-tests where ****p ≤ 0.0001.

Initial development showed that not all EBV sero-positive donors had EBV peptide-reactive T-cell responses, resulting in poor yield of target isolation (**Figure 2A**). To minimize manufacturing failure rate, donors were pre-screened for EBV peptide T-cell responses prior to apheresis, with a minimum criterion of ≥0.08% EBV-peptide-reactive cells needed (**Figure 2B**). Following selection, IFN-γ+ targets expanded 1,000-fold in 18 days of culture, generating approximately 10^10^ EBV VST for cryopreservation into multiple treatment doses (**Figures 2C, D**). Numerous centers directly infuse the peptide-selected IFNγ+ targets as a minimally manipulated cellular product (12); however, this isolates a very low yield (~10^6^) that can only be used for a single patient dose. As well as increasing the EBV VST yield, our subsequent culture process drives an outgrowth of a highly purified central memory population by days 14–18 (**Figure 2E**) containing multifunctional cytokine-secreting cells upon EBV peptide recall (**Figure 2F**). In full-scale manufacture, peptide-derived VST had significantly improved final product viability (**Figure 2G**) and yield for treatment doses (**Figure 2H**) compared to LCL-derived VST.

### 3.2 Differences in immunophenotype between peptide and LCL-derived EBV VST

**Figure 3:**
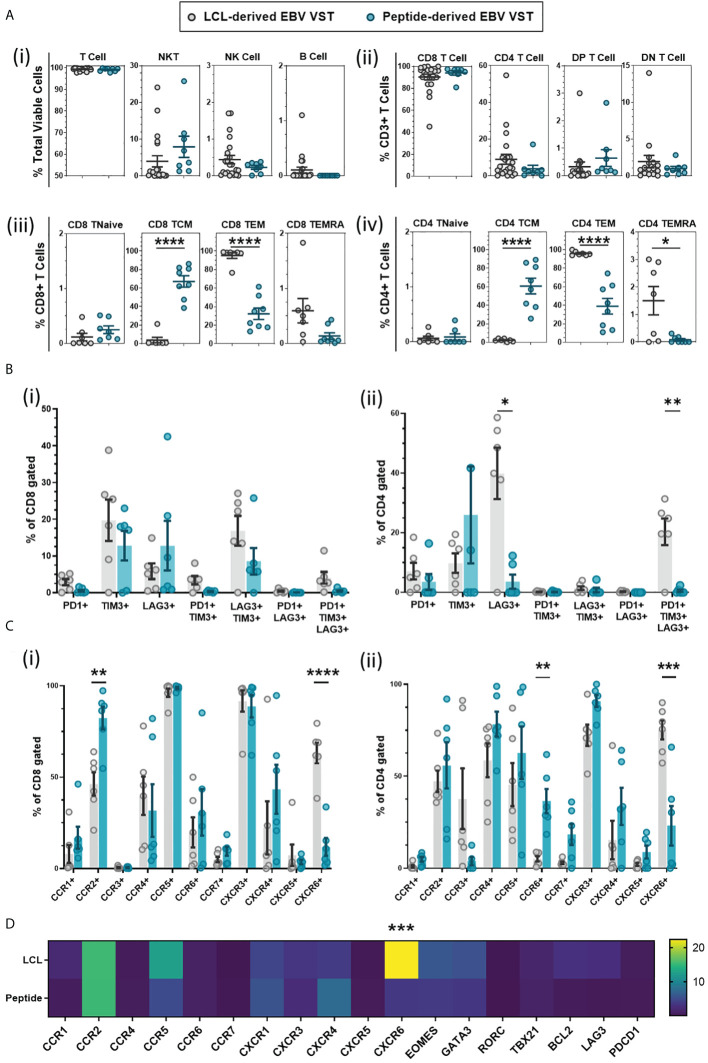
Phenotyping comparison of EBV VST generated using LCL *versus* peptide methods. **(A)** Immunophenotyping of VST at the end of manufacture indicated that there were no significant differences in (i) total lymphocyte populations or (ii) CD4:CD8 content of the products from the LCL group (*n* = 22) and peptide group (*n* = 8). However, there were significant differences in T-cell memory population frequencies between LCL-derived (*n* = 12, memory phenotyping only assessed on LCL banked lines with sufficient material) and peptide-derived EBV VST (*n* = 8), with peptide-derived (iii) CD8 and (iv) CD4 populations demonstrating a consistent central memory phenotype rather than an effector memory profile in LCL-generated final products. **(B)** Final product VST were assessed for T-cell exhaustion through the co-expression of markers PD-1, TIM-3, and LAG-3. While CD8+-gated VST (i) showed a comparable exhaustion marker expression, CD4+-gated VST (ii) of LCL-derived lines had a significantly higher expression of LAG3+ (single expression) and PD1+/TIM3+/LAG3+ (tri-expression) populations compared to peptide-derived lines. **(C)** Lines were also analyzed by flow cytometry for chemokine receptor expression with significant differences in (i) CD8+ cell expression of CCR2, CCR7, and CXCR6 and (ii) CD4+ cell expression of CCR6 and CXCR6 between the two manufacturing methods. **(D)** Transcriptomic analysis confirms a significant upregulation of CXCR6 in LCL-derived VST compared to peptide-derived VST using Taqman low-density array. Data is presented as 2^-ΔΔCT^ ΔΔCT values calculated using housekeeper gene RPLPO of VSTs normalized to naïve T cells isolated from PBMC. Note that only lines with sufficient material were tested for extra phenotyping and transcriptomic analysis in **(B–D)** for the LCL group (*n* = 6) and peptide group (*n* = 6). All data are presented as mean ± SEM. Statistical analysis was done using unpaired *t*-tests (Holm–Šídák for multiple comparisons) with *p ≤ 0.05, **p ≤ 0.01, ***p ≤ 0.001, and ****p ≤ 0.0001.

To compare EBV VST final products between the two manufacturing processes, cryopreserved material from each bank were thawed and assessed for immunophenotype. Due to the differences in stimulation source, markers for potential contaminating cells should be incorporated into the product release criteria. To this end, LCL-derived VST have release criteria of <2% B cells, and since IFN-γ is a potent natural killer (NK) cell cytokine, peptide-derived VST have a release criteria of <10% NK cells. Flow cytometric analysis demonstrated an equivalent frequency of T cell, natural killer-like T cells (NKT cell), NK cell, and B cell populations (**Figure 3A-i**), and both processes primarily generated VST products skewed toward CD8+ T cells (**Figure 3A-ii**). T-cell memory profiling by surface markers (CD62L/CD45RA/CD45RO) within both (**Figure 3A-iii**) CD8+ and (**Figure 3A-iv**) CD4+ compartments revealed a significantly higher percentage of central memory T cells (TCM) in the peptide-derived VST. LCL-derived VST had significantly higher frequencies of effector memory (TEM) and terminal effector compared to peptide derived in both CD8+ and CD4+ compartments.

T-cell activation/exhaustion markers PD-1, Tim-3, and Lag-3 were assessed, and while there were no significant differences between the two groups in CD8+ cells, there was a consistent trend to lower the expression of all exhaustion markers in peptide-generated VST (**Figure 3B-i**). The LCL-derived CD4+ cells had a significantly higher percentage of Lag3+ and PD1+/Tim3+/Lag3+ populations (**Figure 3B-ii**).

Chemokine receptor labeling indicated that CD8+ cells in LCL-derived VST had a significantly lower expression of c-c chemokine receptors (CCR) CCR2 and CCR7 but a higher expression of c-x-c chemokine receptor (CXCR) CXCR6 compared to peptide-derived VST (**Figure 3C-i**). The CD4+ cell expression of CXCR6 was significantly increased in LCL-derived VST and showed a decreased CCR2 and CCR7 expression as well as a significantly lower expression of CCR6 than peptide-derived VST (**Figure 3C-ii**). Increased CXCR6 expression in LCL-derived VST was confirmed at the transcriptome level using TLDA analysis (**Figure 3D**).

### 3.3 Peptide-derived VST have enhanced degranulation/cytokine effector function than LCL-derived VST

**Figure 4:**
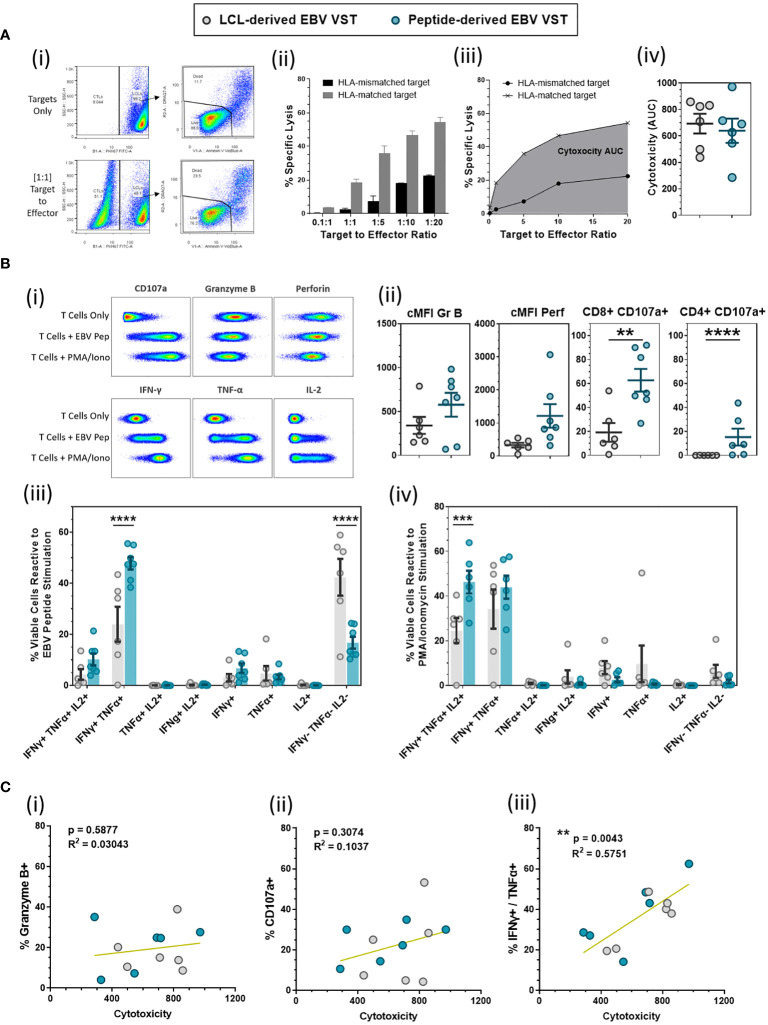
Functional evaluation of LCL-derived *versus* peptide-derived EBV-specific T cells. **(A)** Cytotoxicity of EBV VST made using the LCL and peptide method was compared using a cytotoxicity assay against HLA-matched and HLA mis-matched EBV-infected cell lines (LCL) as targets over numerous target-to-effector ratios (representative examples are shown in i and ii). A single value of overall cytotoxicity (iii) for each VST line was calculated as the AUC for the specific lysis of HLA-matched targets over all T:E ratios, with equivalent cytotoxicity (iv) demonstrated between LCL-derived (*n* = 6) and peptide-derived VST (*n* = 6). **(B)** EBV VST final products were assessed for functional response to EBV peptides compared to positive (PMA/ionomycin) and negative controls (T cells only) as shown in examples of the flow cytometric analysis (i). There was a significant increase in degranulation (CD107a) from both CD8 and CD4 populations in peptide-derived VST (ii). Analysis of multifunctional T-cell subpopulations based on the co-expression of cytokines reveals that peptide-derived VST have a significantly higher frequency of dual IFN-γ+/TNF-α+ cells in response to EBV peptides (iii) and a significantly higher frequency of triple IFN-γ+/TNF-α+/IL-2+ cells in response to PMA/ionomycin stimulation (iv). All data are represented as mean ± SEM. Statistical analysis was done using unpaired *t*-tests (Holm–Šídák for multiple comparisons) with **p ≤ 0.01, ***p ≤ 0.001, and ****p ≤ 0.0001. **(C)** VST lines were assessed for the correlations between cytotoxicity and expression of (i) Granzyme B, (ii) CD107a, and (iii) total IFN-γ+/TNF-α+ (in response to EBV peptide stimulation). Correlations were tested using computed Pearson correlation coefficients where *p* < 0.05 was considered statistically significant.

VST from both banks were tested in numerous functional assays to determine the cytotoxicity, degranulation, and expression of cytokines. Directed killing was measured by cytotoxicity assay co-culturing HLA-matched and HLA-mismatched EBV-presenting LCL targets with VST at increasing target-to-effector ratios (**Figure 4A-i**). Specific lysis at each ratio was normalized to baseline target cell death from target-only controls (**Figure 4A-ii**). The area under the curve (AUC) of HLA-matched target-specific lysis was then calculated for a given VST product to allow a normalized comparison of cytotoxic ability (**Figure 4A-iii**). To this end, both LCL- and peptide-derived EBV VST demonstrated comparable cytotoxicity against infected targets (**Figure 4A-iv**).

EBV VST were also assessed for degranulation and cytokine production in response to EBV peptides (**Figure 4B-i**). While the level of granzyme B and perforin expression upon EBV recall was comparable between the two processes, the frequency of CD8+/CD107a+ cells and CD4+/CD107a+ cells was significantly higher in peptide-derived compared to LCL-derived EBV VST (**Figure 4B-ii**). The percentage of IFN-γ+/TNF-α+ cells was also significantly higher in peptide-derived VST, whereas LCL-derived VST contained a larger proportion of cytokine-null cells upon EBV recall (**Figure 4B-iii**). Furthermore, the percentage of IFN-γ+/TNF-α+/IL-2+ cells in response to PMA/ionomycin was significantly higher in peptide-derived VST, indicating a more potent antiviral cytokine capacity (**Figure 4B-iv**). While cytotoxicity did not correlate with the expression of Granzyme B (**Figure 4C-i**) or CD107a (**Figure 4C-ii**), there was a significant correlation between cytotoxicity and IFN-γ+/TNF-α+ co-expression upon EBV peptide stimulation (**Figure 4C-iii**).

### 3.4 EBV VST are oligoclonal and express known EBV-specific sequences

**Figure 5:**
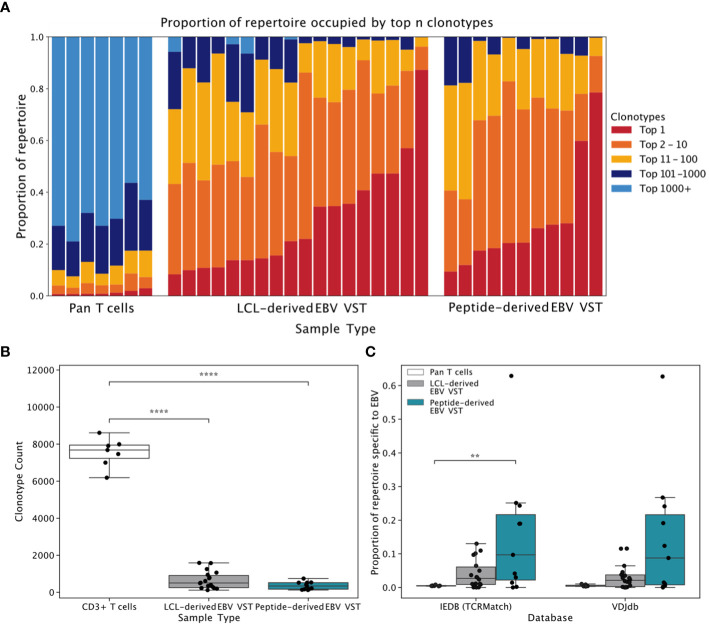
TCR repertoire analysis comparison of EBV VST generated against LCL vs. peptides. **(A)** Stacked bars represent the proportion of the repertoire occupied by the top *n* clonotypes in each product. EBV VST are oligoclonal compared to bulk T cell controls but show substantial variation in clonal diversity between individual products. **(B)** Clonotype counts for UMI size matched repertoires (9542 UMIs) demonstrate that both LCL-derived (*n* = 18 donors) and peptide-derived VST (*n* = 11 donors) had significantly reduced diversity compared to bulk T cells (*n* = 7 donors). Groups were compared using one-way ANOVA and Tukey’s *post-hoc* test. **(C)** The proportion of each repertoire occupied by sequences with an exact CDR3 amino acid sequence match to an EBV-specific TCR in the IEDB (using TCRMatch) and VDJdb databases indicated no significant differences in EBV-specificity between the two groups. Groups were compared using Kruskal-Wallis with Dunn’s *post hoc* test. Only significant comparisons are illustrated. **1.00e-03 < p <= 1.00e-02, ****p <= 1.00e-04.

**Figure 6:**
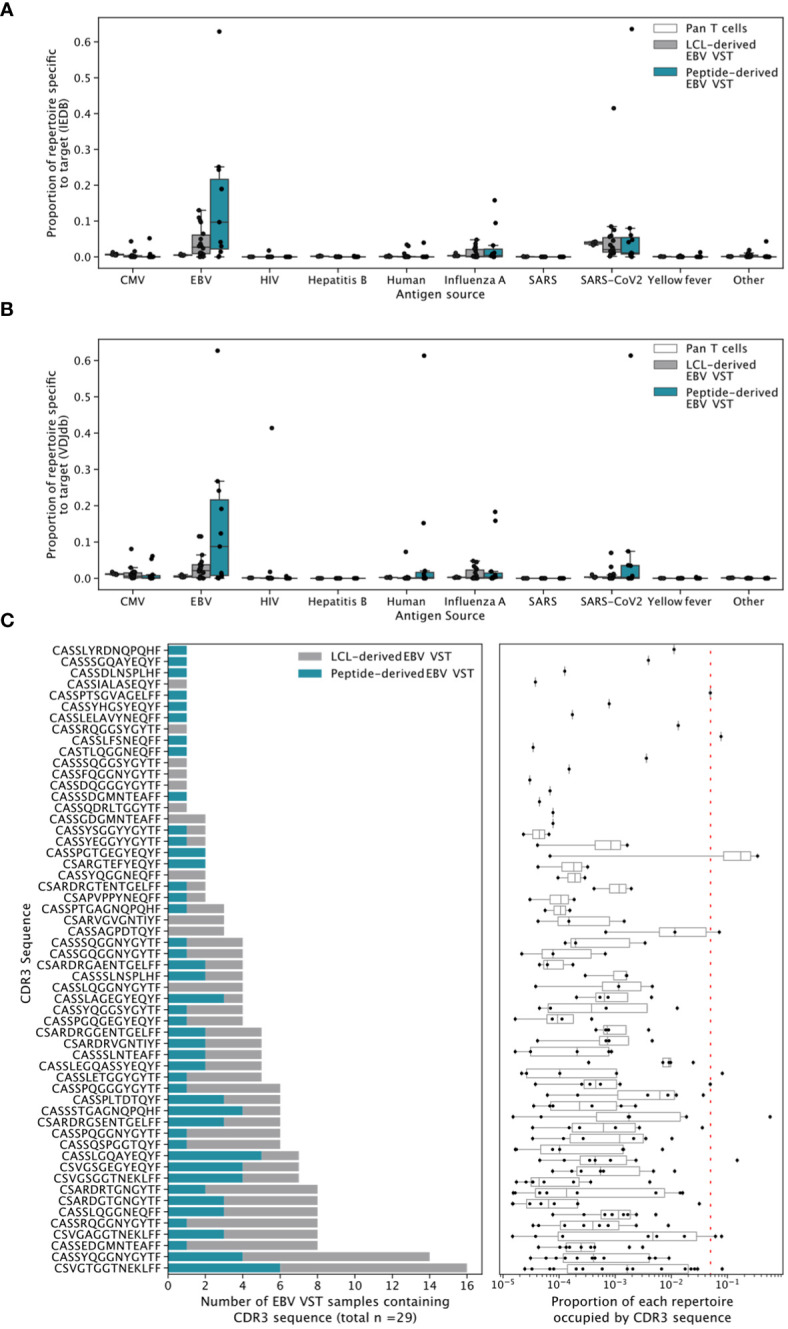
Broadly antigen-specific and public EBV-specific CDR3 sequences in EBV VST. Sequences were annotated with a wide range of antigen specificities by exact CDR3 amino acid match to recorded TCR/epitope entries in **(A)** IEDB and **(B)** VDJdb. EBV-specific sequences appeared to occupy the greatest proportion of the VST repertoires. (C) EBV VST contain 58 public EBV-specific CDR3 amino acid sequences previously identified by Huisman et al. The left-hand panels show the number of all EBV samples which contained the CDR3 amino acid sequence, colored according to LCL vs. peptide manufacture. The right-hand panels show the proportion of each repertoire occupied by the CDR3. Each point represents a single repertoire, and the dashed line represents a proportion of 0.05. The samples were size-matched to 31,233 UMIs.

High-throughput TCRβ sequencing was used to assess clonal diversity. T-cell clonotypes were defined by sharing of the same complimentary determining region-3 (CDR3) amino acid sequence, and the proportion of each T cell product occupied by the top *N* number of clonotypes was quantified (**Figure 5A**). Bulk T cells from donors were used as controls and showed highly diverse TCR repertoires, containing thousands of small clones. In contrast, EBV VST derived by either LCL or peptides were oligoclonal, with individual repertoires dominated by either a single clonotype or between 10 and 100 clonotypes. Overall, clonotype count (**Figure 5B**) was significantly lower in both EBV VST groups compared to bulk T cells. When two databases of known TCR/antigen binding (IEDB and VDJdb) were queried, a significantly greater proportion of the repertoire in peptide-derived VST comprised sequences annotated as EBV-specific compared with bulk T cells (**Figure 5C**). Sequences with a wide range of other known antigen specificities were also identified, although EBV-specific sequences comprised the greatest proportion of VST (**Figure 6A**).

Public (or shared) T-cell responses to EBV have previously been observed. To explore such responses in EBV VST, we searched for the 98 EBV-specific public CDR3 sequences described in Huisman et al. (27). We identified 58, with 41 present in more than one sample. The most shared CDR3 (specific to the BLMF1 antigen) was found in half of all VST (15/29; **Figure 6B**). These public CDR3 sequences were of low abundance in individual repertoires, suggesting that the majority of expanded responses are private to the donor. The likelihood of a CDR3 sequence to occur by chance in the repertoire, or its generation probability (Pgen), is affected by biases in the VDJ recombination process, such as gene usage and rates of nucleotide insertion and deletion. Mean Pgen values were significantly lower in peptide-derived VST compared to both bulk T cells and LCL-derived VST (**Supplementary Figure S3A**). When weighted by clonotype frequency, the mean Pgen values in LCL-derived VST were not significantly different from those in peptide-derived ones (**Supplementary Figure S3B**). This effect is driven by similarly low Pgen values in the largest clonotypes for both VST groups and higher Pgen values in less expanded LCL-derived clonotypes (**Supplementary Figure S3C**).

### 3.5 Peptide-derived VST demonstrate a higher reactivity to EBNA antigens

**Figure 7:**
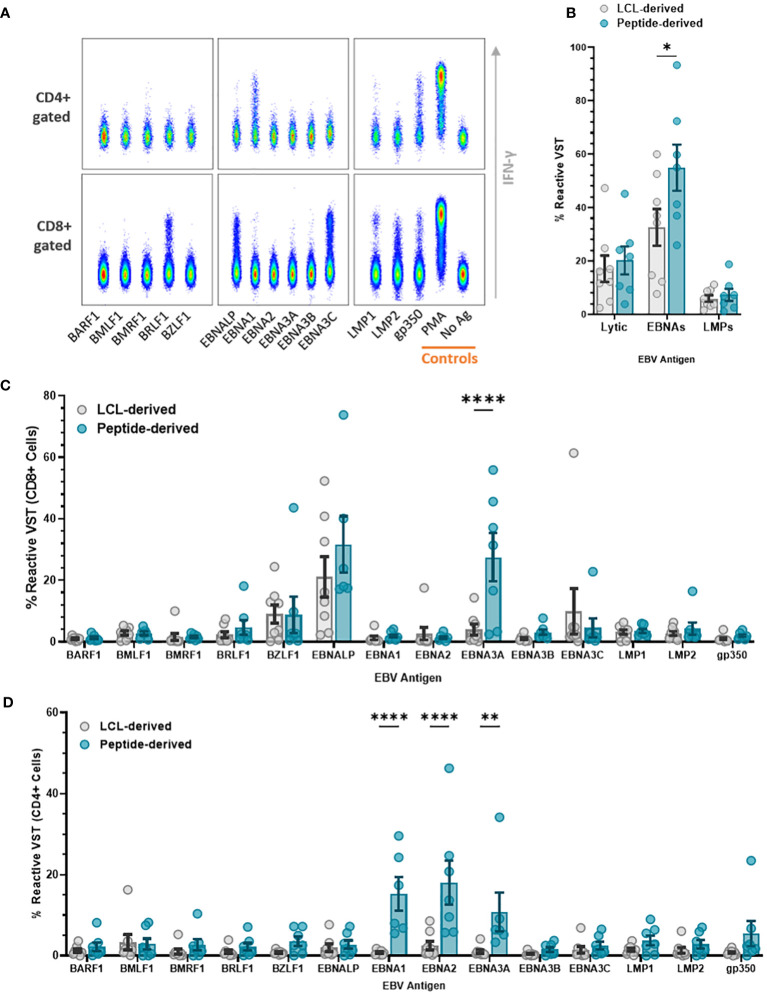
Antigen specificity comparison of EBV VST generated against LCL vs. peptides. EBV VST were assessed for antigen specificity by stimulation with individual EBV antigen pepmix pools followed by intracellular staining of reactive antigen-specific VST with effector markers. **(A)** Flow cytometric analysis of representative EBV VST shows IFN-γ+ reactive VST to EBV antigens, with positive control (PMA) and negative control (no ag). **(B)** Peptide-derived T cell products were more targeted toward EBNA antigens than LCL-derived products. Individual antigen specificity is compared between the two groups within the **(C)** CD8+ and **(D)** CD4+ compartments. Data is represented as mean ± SEM, and statistical analysis was done using unpaired *t*-tests (Holm–Šídák for multiple comparisons) with *p ≤ 0.05, **p ≤ 0.01, and ****p ≤ 0.0001.

The difference in antigen type used meant that specificity to individual EBV antigens varied between the two banks. Reactivity to individual EBV antigens—BARF1, BMLF1, BMRF1, BRLF1, BZLF1, EBNA-LP, EBNA1, EBNA2, EBNA3A, EBNA3B, EBNA3C, EBNA3C, LMP1, LMP2, and gp350/340—was assessed by the production of effector markers (**Figure 7A**). Total VST reactivity (**Figure 7B**) shows targeting toward EBNA antigens, significantly higher in peptide than LCL-derived VST. Within the CD8 compartment (**Figure 7C**), both VST products demonstrated a conserved antigen reactivity mainly to BZLF1, EBNA-LP, EBNA-3A, and EBNA-3C. Peptide-derived VST had a significantly higher CD8+ reactivity to EBNA3A compared to LCL-derived VST. Peptide VST also had a significantly higher CD4+ cell reactivity (**Figure 7D**) compared to LCL VST for antigens EBNA1, EBNA2, and EBNA3A.

## 4 Discussion

Adoptive transfer of allogeneic EBV VST for the treatment of post-transplant EBV-induced lymphoma has been one of the pioneering T-cell therapies to demonstrate clinical success. As technologies for antigen-specific T-cell therapies advance in the field, characterization is essential to understand the effects of T-cell generation methodology. In the current study, we compared EBV VST generated using LCL stimulation *versus* peptide-mediated selection and expansion using comprehensive profiling of phenotype, functionality, specificity, and clonal composition.

Generation of EBV VST has conventionally used EBV-transformed LCL to drive the expansion of EBV VST due to their strong antigen-presenting capacity and expression of costimulatory molecules. We have previously described the establishment of an EBV VST bank manufactured by LCL stimulation (18), with multiple follow-up studies demonstrating clinical efficacy in refractory or relapsed PTLD patients (7, 29).

A potential issue with the LCL process involves the biosafety risk around the culture of EBV-infected LCL. While formalin fixation of LCL has been used to reduce the biosafety risk (30), serial passage of LCL is associated with accumulation of non-silent mutations which could induce non-specific T-cell responses (31). We introduced an alternative approach using EBV peptide pools as the antigenic stimulation. Through a combination of the peptide-induced IFN-γ selection of EBV VST followed by expansion using entirely closed-system processes and GMP-compliant reagents, we identified several practical advantages, including reduced process manipulations, reduced hands-on time, and shorter culture duration (see **Table 3**). Reducing steps minimizes risk to introduced contaminants and releases benefits in terms of staff time and clean-room facility costs, while automation also standardizes processing for robust product manufacture. Additionally, we showed significantly improved viability in peptide-derived VST in comparison to LCL-derived VST, which likely reflects the shorter culture duration. Despite a high upfront cost of the peptide process, the 10-fold increase in treatment doses produced results in a more cost-effective manufacturing process (33) compared to LCL manufacture (32). Moreover, the peptide process is a readily translatable platform to generate T cells targeting other antigens or viruses (34).

**Table 3:** Comparison of EBV VST manufacturing methods.

Method	Advantages	Disadvantages
LCL repeated stimulation	• Established clinical response rate (60%–90%) • VST recognize a broad range of EBV latent and lytic peptides • Can derive VST from EBVseropositive and seronegative donors • Low upfront reagent/ material cost per manufacturing run (~£3615, 32). However, the low yield (mean = 9 doses) gives a final cost per dose (£401)	• Long protocol duration (8–12 weeks), higher facility cost • VST yield is limited by the availability of high numbers of LCLs • VST contain a smaller central memory T-cell pool • Multiple stimulation rounds entail many culture manipulations and higher personnel and facility costs • Biosafety risk with the use of live virus to generate LCLs
Peptide-mediated IFN-γ selection and expansion	• Shorter protocol duration (18 days) and reduced facility cost. • All closed-process isolation and culture • Improved viability and degranulation/cytokine effector capacity • Significantly greater yield per manufacturing process • Can tailor the process to a particular EBV antigen/peptide mix • Minimal manipulations and reduced personnel cost	• VST are restricted to the recognition of consensus EBV peptide pools • Restricted to EBV-seropositive donors for manufacturing • IFN-γ selection protocol can isolate NK cells/ NKT cells => require stringent final product release criteria • High upfront reagent/material cost per manufacturing run (~£22,000, 33). However, the high yield (mean = 84 doses) gives a low final cost per dose (£262)

EBV VST from both processes demonstrated a highly enriched T-cell product containing majorly CD8+ cells with a small population of CD4+ cells. Importantly, peptide-derived VST were significantly skewed toward central memory. Conversely, LCL-derived VST were more differentiated to effector memory, which may reflect repeated antigenic stimulation. Given the increased proliferation capacity of central memory T cells, this may suggest that peptide-derived VST will persist longer upon adoptive transfer (35). Tracking of gene-marked cytomegalovirus (CMV) VST from purified central memory versus effector memory populations adoptively transferred to non-human primate macaques demonstrated the persistence of TCM clones for up to 344 days, whereas TEM clones failed to persist >7 days post-infusion (36). Similarly, tumor-specific CD8+ cells skewed to TCM phenotype exhibited enhanced tumor clearance compared to TEM in adoptive transfer experiments in mice (37), suggesting that lymphoid homing T cells may be optimal for adoptive immunotherapy.

EBV lymphomas are commonly diffuse with extranodal involvement in numerous sites, including kidney, liver, lung, spleen, gastrointestinal tract, and central nervous system (38). It is therefore crucial that peripherally infused EBV VST can migrate and infiltrate within these sites in a tumor-targeted manner. Chemokine receptor expression was largely consistent in both; however, peptide-derived VST had significantly higher levels of CCR2 and CCR7. The CCR2 ligand CCL2 has been observed to be upregulated in EBV lymphoma lines (39); therefore, higher CCR2 expression indicates an increased capacity for peptide VST to migrate to and infiltrate within the tumor microenvironment (40). The high expression of CXCR6 on LCL-derived VST is consistent with a previous study (41) where CXCL16 secretion from nasopharyngeal carcinoma recruited CXCR6^high^ T cells to the tumor site. While a genome-wide association study identified a positive correlation between LCL CXCL16 expression and EBV copy number (42), it is not known if CXCR6 is required for the effective trafficking of VST to EBV PTLD tumor sites. Since EBV+ lymphomas secrete high quantities of CCL3, CCL4, CCL22, and CXCL10 compared to EBV- lymphoma lines (39), the high expression of CCR5, CCR4, and CXCR3 on both EBV VST groups may indicate complementary/alternate axes of migratory mechanisms.

In terms of potency, peptide-derived VST had equivalent cytotoxic capacity to LCL-derived VST, which have already demonstrated their efficacy in patient treatment. After antigen recall, peptide-derived VST had increased CD107a expression and higher frequency of multifunctional cytokine-producing populations, indicating that these cells have enhanced degranulation and co-stimulatory effector functions. Interestingly, cytotoxicity against EBV-infected target cells positively correlated with the percentage of IFN-γ+/TNF-α+ cells reactive to EBV peptides. This peptide antigen recall assay could therefore be used in quality control as a surrogate assay to replace cytotoxicity assays which require the culture of autologous virus-infected target cells.

An analysis of the TCRβ repertoire showed a substantial variation in clonotype size between VST, with certain isolates dominated by individual CDR3 sequences. In some products, a large proportion of the repertoire was confirmed to contain sequences which are known to be EBV-specific in public databases. Many previously identified EBV-specific public CDR3s were also identified, although these were present at very low frequencies in most VST. The mean generation probability of sequences is also reduced in EBV VST compared with bulk T cells, suggesting that TCRs were not present purely by chance but appear to have been actively selected. Even small clonotypes in peptide-derived samples have low Pgen values, whereas in LCL-derived VST these were increased, indicating the presence of less targeted cells in these products. The majority of the repertoire in EBV VST is comprised of a restricted number of highly expanded clonotypes, which are less commonly occurring and represent private EBV responses in the donor individuals. Peptide-derived VST trended toward a higher proportion of the repertoire consisting of sequences annotated as EBV-specific public databases than LCL-derived VST, potentially reflecting the different antigen sources used. It is likely that there is a significant overlap in the EBV peptides used for stimulation and those used in the peptide major histocompatibility complex (MHC) multimer experiments that contribute to TCR/epitope database curation. The range and density of antigens presented by LCL are likely to be more diverse and vary according to donor and HLA type (43), potentially leading to greater inter-batch variability and more limited overlap with epitopes present in online databases. Interestingly, EBV VST from both processes had a small proportion of the repertoire that matches to influenza A virus (IAV) and severe acute respiratory syndrome coronavirus 2 (SARS-CoV-2) sequences. All sequenced EBV VST were manufactured prior to 2019; therefore, the annotation of SARS-CoV-2-specific sequences found in this study may represent cross-reactivity with pre-existing immunity to other human coronaviruses (44) or sequence homology in some EBV, IAV, and SARS-CoV-2 epitopes (45).

Specific antigen reactivity demonstrated that EBV VST were mainly targeted toward EBNA-LP, EBNA-3A, EBNA-3C, and BZLF1 antigens. Total reactivity to EBNAs and particularly EBNA-3A was higher in peptide-derived VST. CD4+ T cells within peptide-derived VST were also more reactive to EBNA-1, EBNA-2, and EBNA-3A. Considering that CD4+ responses are negligible for all other tested antigens, this indicates that helper cells expanded through the peptide process have greater functional capacity for antigen recall.

This study describes the development and implementation of a new manufacturing process to generate EBV VST for clinical use with significant benefits in terms of product yield, cost-effectiveness, and closed/automated processing. While clonal diversity and cytotoxicity was comparable, peptide-derived VST demonstrated enhanced degranulation, cytokine production to a broad range of EBV latent antigens, and a dominant central/effector memory status which may improve persistence and targeted tumor clearance in EBV lymphoma patients. The efficacy of these T-cell products will be followed as they continue to be supplied on a compassionate basis under “Specials” license to patients worldwide.

## Data availability statement

The datasets presented in this study can be found in online repositories. The names of the repository/repositories and accession number(s) can be found below: https://www.ebi.ac.uk/ena, PRJEB69214.

## Ethics statement

The studies involving humans were approved by Scottish National Blood Transfusion Service Ethics Committee. The studies were conducted in accordance with the local legislation and institutional requirements. The participants provided their written informed consent to participate in this study.

## Author contributions

RC: Conceptualization, Data curation, Formal analysis, Funding acquisition, Investigation, Methodology, Project administration, Resources, Software, Supervision, Validation, Visualization, Writing – original draft, Writing – review & editing. CS: Data curation, Investigation, Methodology, Visualization, Writing – original draft, Writing – review & editing. LS: Data curation, Methodology, Writing – original draft, Writing – review & editing. GC: Conceptualization, Data curation, Formal analysis, Funding acquisition, Investigation, Methodology, Project administration, Resources, Software, Supervision, Writing – original draft, Writing – review & editing. MB: Data curation, Supervision, Writing – review & editing. DM: Data curation, Project administration, Resources, Supervision, Writing – review & editing. CM: Data curation, Supervision, Validation, Writing – review & editing. SI: Investigation, Methodology, Writing – review & editing. AH: Methodology, Resources, Writing – review & editing. SZ: Investigation, Project administration, Resources, Writing – review & editing. CM: Project administration, Resources, Writing – review & editing. MV: Investigation, Methodology, Resources, Writing – review & editing. GG: Formal analysis, Investigation, Methodology, Project administration, Writing – review & editing. NM: Funding acquisition, Project administration, Resources, Supervision, Validation, Writing – review & editing. MT: Conceptualization, Funding acquisition, Investigation, Methodology, Project administration, Resources, Supervision, Writing – review & editing. JC: Methodology, Project administration, Resources, Software, Supervision, Validation, Visualization, Writing – original draft, Writing – review & editing, Conceptualization, Data curation, Formal analysis, Funding acquisition, Investigation. AF: Conceptualization, Data curation, Formal analysis, Funding acquisition, Investigation, Methodology, Project administration, Resources, Software, Supervision, Validation, Visualization, Writing – original draft, Writing – review & editing.
