# Supplementary material for: EBV T-cell immunotherapy generated by peptide selection has enhanced effector functionality compared to LCL stimulation
Source: Front Immunol. 2024 Jul 1;15:1412211. doi: 10.3389/fimmu.2024.1412211 (PMC11246990; doi:10.3389/fimmu.2024.1412211)

Supplementary Material

# Supplementary Methods

# *Flow Cytometry Analysis*

# Flow cytometric data was analysed using FlowJo v10.6.1 (BD Biosciences). All data analysis was subject to an initial gating strategy (Supplementary Figure 1A) to gate on lymphocytes for starting material or all cells for final product material on the basis of FSC/SSC properties. This population was sequentially gated on singlets, and viable cells by negative expression of dead cell dye (DRAQ7 or FVD).

# For surface immunophenotyping of final product material, viable cells were initially quantified for percentage expression of the following populations: T cells (CD3+/CD56-), NKT cells (CD3+/CD56+), NK cells (CD3-/CD56+) and B cells (CD19+). Gated CD3+ cells were then gated for T cell subpopulations: CD8 T cells (CD8+/CD4-), CD4 T cells (CD4+/CD8-), double positive (DP) T cells (CD4+/CD8+) and double negative (DN) T cells (CD4-/CD8-). Within both CD4+ and CD8+ gated, cells were then analysed for T cell memory populations (Supplementary Figure 1B): naïve T cells (TNaive: CD62L+/CD45RA+), central memory T cells (TCM: CD62L+/CD45RO+), effector memory T cells (TEM: CD62L-/CD45RO+), terminally differentiated T cells (TEMRA: CD62L-/CD45RA+). For T cell exhaustion phenotyping, single expression and co-expression of activation/exhaustion markers LAG-3, PD-1, and TIM-3 were quantified as percentage frequency. For chemokine receptor profiling, gates for acquisition of chemokine receptor expression were placed according to FMO controls.

# For intracellular cytokine/degranulation analysis, treatment conditions were gated according to negative control. Degranulation was analysed on the basis of percentage of CD107a+ cells, and corrected MFI for Granzyme B and Perforin expression. Cytokine reactivity was measured on the basis of co-expression or singular expression of cytokines IFN-γ, TNF-α and IL-2 (Supplementary Figure 1C).

# *TCRβ next generation sequencing*

# *RNA extraction & cDNA preparation*

# RNA was extracted using Quick-RNA MiniPrep Plus Kit (Zymo Research) according to manufacturer’s guidelines, with 15 minutes DNAse I treatment on column to minimize genomic DNA contamination. cDNA synthesis optimised for TCRβ amplification was performed as outlined19 in which 8µL RNA was added to 2µL of 20µM BC1R primer and incubated for 2 minutes at 72°C followed by 3 minutes at 42°C. Reactions were then set up for cDNA synthesis by incubating the RNA mix above with the following mastermix: 2µL 10µM SmartNNN template switch oligonucleotide, 2µL 10mM dNTP mix (ThermoFisher Scientific, 0.5µL 20mM DTT, 2µL [100U/µL] SMARTscribe reverse transcriptase, 0.5µL [40U/µL] RNAse inhibitor and 4µL [5X] First strand buffer (all Takara Bio). The SMARTNNN primer allows cDNA synthesis with 5’ template switching and introduction of unique molecular identifiers (UMIs) to quantify starting cDNA molecules, prevent PCR amplification bias and allow for error correction. Samples were incubated at 42°C for 45 minutes, followed by 70°C for 10 minutes to terminate the reaction. Samples were finally incubated with 1µL [5U/µL] Uracyl DNA Glycosylase (New England BioLabs) at 37°C for 10 minutes to prevent exchange of unique molecular identifier sequences during subsequent amplification steps.

# *TCRβ amplicon generation & next generation sequencing*

# Indexed forward primers composed of the SMART oligo sequence and a P7 Illumina tag, and a reverse primer within the TCR constant region fused to a P5 Illumina tag, were used to allow for later Illumina sequencing (Supplementary Figure 2). Fresh cDNA (2µL) was added to mastermix: 2µL 10µM Smart_stepout 1 primer, 2µL 10µM BC2R primer, 10µL [2X] Phusion Flash high fidelity mastermix (ThermoFisher Scientific) and 4µL nuclease-free water. Samples were incubated at 98°C for 2 minutes; followed by 18 amplification cycles at 98°C for 5 seconds, 68°C for 10 seconds and 72°C for 15 seconds. Samples were then incubated at 72°C for 4 minutes for final elongation. For the second amplification 2µL of the first PCR product was added to the following mastermix: 2µL 10µM P7-SMART-Index, 2µL 10µM P5-BCJ primer, 10µL [2X] Phusion Flash high fidelity mastermix and 4µL nuclease-free water. PCR conditions were as for the first amplification but only 12 cycles performed. All primers used were custom generated DNA oligomers from IDT as detailed in Table 2. Second PCR products were assessed for purified TCRβ amplification using gel electrophoresis, and stored at -20°C prior to pooling/purification using Monarch PCR Gel Extraction Kit (New England BioLabs). 2 x 300bp sequencing of libraries on the Illumina MiSeq platform was performed by GeneWiz using custom read primers: TCR_read 1, TCR_read2 and TCR Index with LNA modifications (Table 2).

# Supplementary Figures and Tables

**Supplementary Figure 1**. Flow cytometry analysis gating strategies. **(A)** All flow cytometric assays analyses were subject to an initial gating strategy to firstly gate on lymphocytes using FSC vs SSC, sequentially gating on single cells (doublets exclusion) using FSC-A vs FSC-H, and finally to gate on live cells (dead cell exclusion) using dead cell dye DRAQ7 or fixable viability dye eFluor780. **(B)** Surface phenotyping using exemplar stained PBMCs identified the following lymphocyte populations: NK cells (CD3-/CD56+), NKT cells (CD3+/ CD56+), CD4 T Cells (CD3+/ CD4+) and CD8 T cells (CD3+/ CD8+). Both CD4 T cells and CD8 T cells were then sequentially gating for T cell memory subpopulations as follows: TNaive (CD62L+/ CD45RA+), TCM (CD62L+/CD45RO+), TEM (CD62L-/CD45RO+) and TEMRA (CD62L-/CD45RA+). **(C)** Intracellular cytokine analysis using PBMCs stimulated with PMA/ionomycin identified singular and multiple cytokine-secreting VST by initial gating of viable cells for expression of IL-2, and each IL-2 population analysed for expression of IFN-γ vs TNF-α. **(D)** Donor pre-screen to test for eligible donors for peptide bank manufacture was gated as above on lymphocytes/ singlets/ viable cells, followed by gating on CD3+ IFN-γ+ cells (gate set according to negative no antigen control).

**Supplementary Figure 2**. TCRβ amplification and sequencing strategy.

14bp UMIs were incorporated during cDNA synthesis by the use of SMARTNNN oligos. This was followed by a two-step PCR amplification process, with a step-out PCR at the 5’ end and a nested PCR at the 3’ end of the targeted TCRβ region. The addition of Illumina tags during the second PCR reaction allows for sequencing with custom primer. Barcoded forward primers were used to demultiplex samples. Reactions were carried out in duplicate post RNA extraction.

**Supplementary Figure 3**. Generation probability of TCRs in EBV VST generated against LCL vs peptides.

Generation probabilities for CDR3 sequences from each repertoire were calculated using OLGA. **(A)** Mean generation probabilities in peptide-derived VST were significantly lower than for both bulk T cells and LCL-derived EBV VST when only unique CDR3s were considered. **(B)** When mean generation probability was weighted by clonotype frequency, both VST groups but both had significantly lower values than observed in the bulk T cells. **(C)** Mean generation probability for each repertoire, split by size of clonotype, unweighted suggests that clonotypes of all sizes in peptide-derived EBV VST have reduced generation probabilities. Samples were subset to 9542 UMIs in all cases. CDR3 sequences with a generation probability of 0 were excluded. Groups were compared using Kruskal-Wallis with Dunn’s post hoc test. Only significant comparisons are illustrated.

**Supplementary Figure 1**.


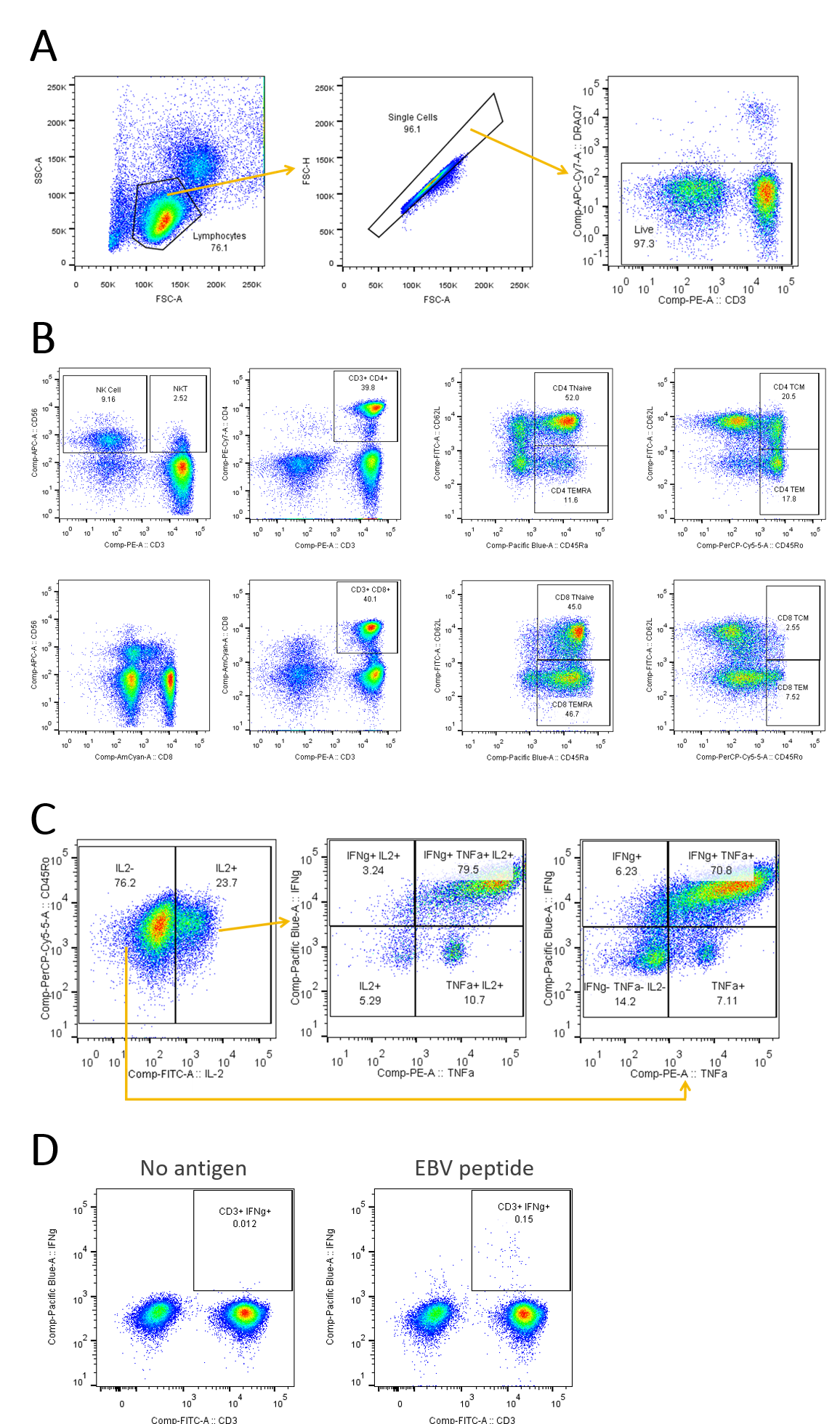


**Supplementary Figure 2**.

**Supplementary Figure 3.**


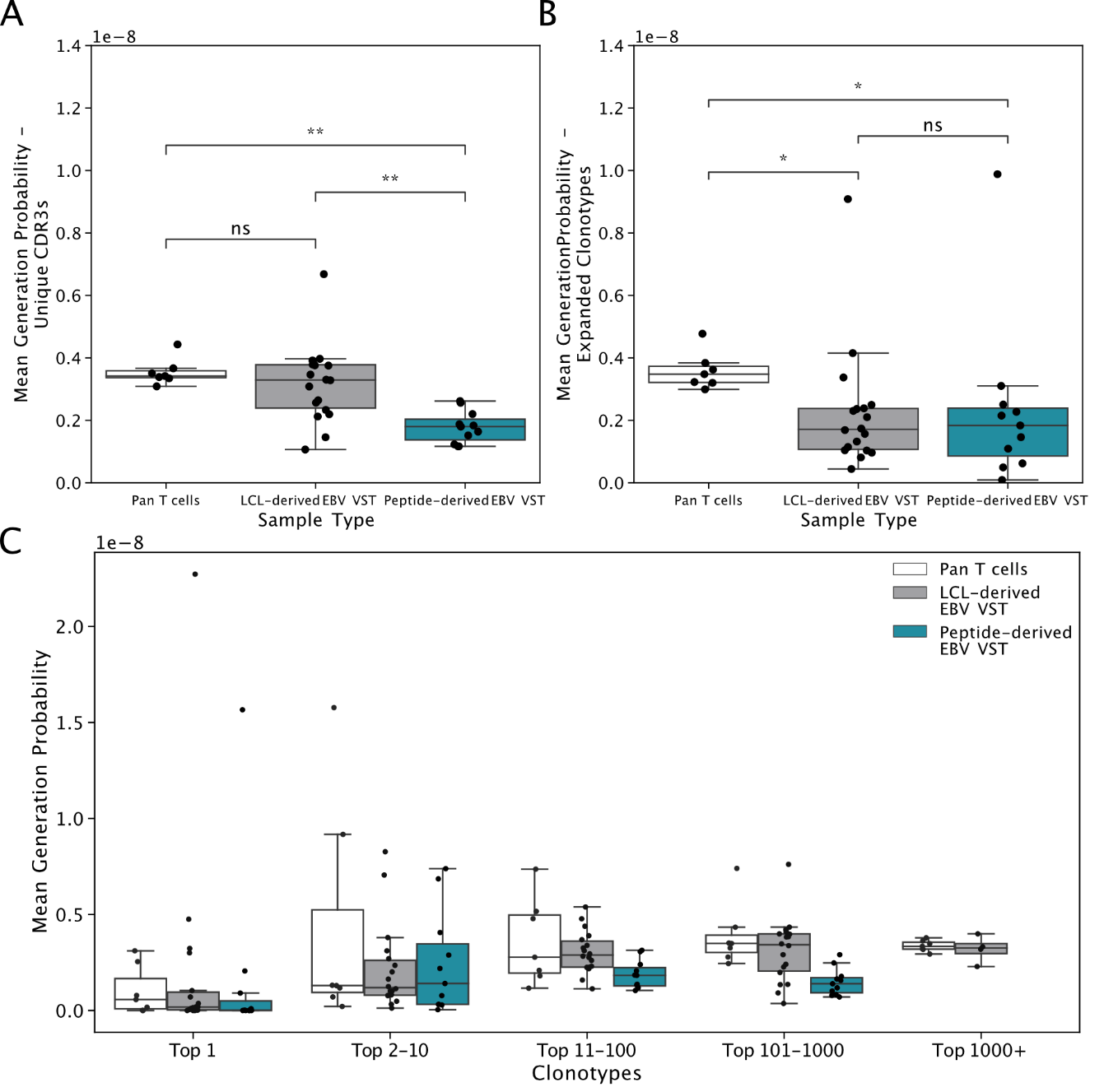

Supplement: Supplementary file 1 [file DataSheet_1.docx]
